# Comparison of severe acute respiratory illness (sari) and clinical pneumonia case definitions for the detection of influenza virus infections among hospitalized patients, western Kenya, 2009‐2013

**DOI:** 10.1111/irv.12382

**Published:** 2016-03-23

**Authors:** Caroline Makokha, Joshua Mott, Henry N. Njuguna, Sammy Khagayi, Jennifer R. Verani, Bryan Nyawanda, Nancy Otieno, Mark A. Katz

**Affiliations:** ^1^ Kenya Medical Research Institute (KEMRI)KisumuKenya; ^2^ Centers for Disease Control and PreventionNairobiKenya; ^3^ Centers for Disease Control and Prevention (CDC)AtlantaGAUSA

## Abstract

Although the severe acute respiratory illness (SARI) case definition is increasingly used for inpatient influenza surveillance, pneumonia is a more familiar term to clinicians and policymakers. We evaluated WHO case definitions for severe acute respiratory illness (SARI) and pneumonia (Integrated Management of Childhood Illnesses (IMCI) for children aged <5 years and Integrated Management of Adolescent and Adult Illnesses (IMAI) for patients aged ≥13 years) for detecting laboratory‐confirmed influenza among hospitalized ARI patients. Sensitivities were 84% for SARI and 69% for IMCI pneumonia in children aged <5 years and 60% for SARI and 57% for IMAI pneumonia in patients aged ≥13 years. Clinical pneumonia case definitions may be a useful complement to SARI for inpatient influenza surveillance.

## Introduction

Many sentinel surveillance systems for hospitalized influenza, particularly in resource‐poor settings, have focused on identifying cases of severe acute respiratory illness (SARI) 1, 2 In 2013, the WHO published a SARI case definition for use in global influenza surveillance (Table 1).^3^ Although the SARI case definition has been widely used for surveillance, policymakers and clinicians are more familiar with pneumonia, rather than SARI, as a clinical respiratory syndrome that causes morbidity and mortality.

**Table 1:** World Health Organization case definitions for severe acute respiratory illness (SARI) for all ages, and clinical pneumonia in children aged <5 years and clinical pneumonia in patients aged ≥13 years

Definition title, age	Definition criteria
SARI‐ WHO 2013,^24^ For all ages	An acute respiratory infection with: History of fever or measured fever of ≥38^°^C And cough With onset within the last 10 days And requires hospitalization
Integrated Management of Childhood Illnesses (IMCI) Pneumonia 11 For children aged 2 months to 5 years	Cough or difficult breathing with: Fast breathing 2‐ <12 months‐ ≥ 50 breaths/min 12‐59 months‐ ≥ 40 breaths/min 5–12 years‐ ≥ 30 breaths/min 13 years and above‐ ≥ 20 breaths/min or any one of: Any danger signs (unconscious, convulsing, unable to breastfeed, vomiting everything) Chest indrawing Stridor when calm.
Integrated Management of Adolescent and Adult Illnesses (IMAI) Pneumonia 12 For patients aged ≥13 years	Cough or difficult breathing with any two of: Fast breathing Chest pain Night sweats

Policy statements by important global health policymakers have reflected the fact that the clinical syndrome of pneumonia, rather than SARI, is a priority for global health. The fourth UN Millennium Development Goal, which focuses on reducing child mortality, specifically describes the need to improve “prevention and case management of pneumonia.”.^4^ In addition, GAVI (The Vaccine Alliance) describes that its primary mission—”to save children's lives and protect people's health by increasing access to immunization in poor countries”—is largely targeted to these MDGs, including reducing childhood pneumonia through immunization.^5^ The Bill & Melinda Gates Foundation's “pneumonia” strategy focuses on “the most prevalent causes of childhood pneumonia—the pneumococcus, influenza, and RSV”.^6^ In 2013, Unicef and WHO outlined their joint Integrated Global Action Plan for Pneumonia and Diarrhoea—a plan to end preventable child deaths from pneumonia and diarrhea by 2025.^7^ The term SARI does not appear in any of these documents. In addition, lack of familiarity with SARI among clinicians is a major challenge for SARI surveillance^8^

The World Health Organization has published case definitions for the detection of pneumonia in clinical field settings (Table 1). While the guidelines were developed for clinical purposes, they have been used for both clinical management and surveillance.^9^, ^10^ The Integrated Management for Childhood Illnesses (IMCI) guidelines outline pneumonia case definitions for children aged <5 years,^11^ and the Integrated Management of Adolescent and Adult Illness (IMAI) guidelines define pneumonia in patients aged ≥13 years.^12^

We evaluated the utility of pneumonia case definitions as compared to SARI case definitions for surveillance of influenza by comparing the sensitivity, specificity, and other surveillance parameters of the 2013 WHO SARI definition to the IMCI and IMAI pneumonia definition for the detection of influenza in hospitalized patients with acute respiratory illness.

## Methodology

The Kenya Medical Research Institute (KEMRI) in collaboration with the US Centers for Disease Control and Prevention (CDC) has been carrying out hospital‐based disease surveillance at Siaya County Referral Hospital (SCRH) since 2009. SCRH is located in rural western Kenya, in the Karemo Division of Nyanza Province. Karemo has an approximate population of 80,000 people.^13^ It is culturally homogenous and almost entirely rural. The area is endemic for malaria with a high child mortality rate (212 deaths per 1000 live births in 2008) 14 and a high HIV prevalence (14% in 2008).^15^ In 2006–2008, the burden of acute lower respiratory illness—calculated using a modified version of the IMCI pneumonia case definition—was 0·36 episodes per year for children aged <5 years and 0·067 episodes per year for persons aged ≥5 years.^9^

At SCRH, we conducted inpatient surveillance for acute respiratory illness (ARI), which we defined as hospitalization with cough or difficult breathing or pleural chest pain with onset in the last 14 days. Trained surveillance officers collected demographic and clinical information, and nasopharyngeal (NP) and oropharyngeal (OP) specimens from ARI patients. Specimens were combined into a single viral transport media and tested by real‐time reverse transcription–polymerase chain reaction (rRT‐PCR) for influenza A and B viruses. Influenza A‐positive samples were analyzed further into subtypes AH1N1 pandemic, AH1N1 seasonal, and H3N2, as previously described.^16^ The WHO‐recommended pneumonia and SARI case definitions are defined in Table 1.

We analyzed data from 2009 to 2013 using stata ^®^ version 11·0 (Statacorp, college station, TX, USA); we used univariate analysis methods, including proportions, to describe demographic, clinical, and laboratory characteristics of patients. Chi‐square tests were used to compare clinical and demographic characteristics of ARI patients with and without rRT‐PCR results. For each of the case definitions (SARI, IMCI, and IMAI), we calculated sensitivity, specificity, positive and negative predictive values, and 95% confidence intervals (CI) for the detection of rRT‐PCR‐confirmed influenza virus.

Surveillance was approved by the KEMRI Ethics Review Committee and Scientific Steering Committee (KEMRI SSC no. 1801) and the CDC Institutional Review Board (CDC IRB no. 3308). Written informed consent was obtained from all participants, parents, or legally authorized representatives.

## Results

Overall, 4715 children aged <5 years were enrolled, for whom 3833 (82%) had laboratory results; 882 (18%) samples were not tested because they did not meet laboratory standards for testing. Age and gender distributions were similar among those tested and not tested for influenza virus infection. Among children aged <5 years, there were less deaths among those tested [3% (104/3883)] compared to those not tested [20% (176/882)] (*P < 0·001*).

Among those tested, 229 (6%) were positive for influenza virus infections: 157 (69%) were influenza A, 73 (31%) were influenza B, and 1 (0·4%) was positive for both influenza A and B. A total of 1255 patients aged ≥13 years were enrolled, including 1150 (91%) with laboratory results. Of these, 128 (11%) were positive for influenza virus, of which 102 (80%) were influenza A, 27 (21%) were influenza B, and 1 (0·8%) was positive for both influenza A and B (Table 2). Among patients aged <5 years, 1998 (52%) met both the SARI and IMCI case definitions, 987 (26%) met the SARI but not the IMCI case definition, 520 (14%) met the IMCI but not the SARI case definition, and 328 (9%) did not meet either case definition. Among those children aged ≥13 years, 356 (31%) met both the SARI and the IMAI case definitions, 196 (17%) met the SARI but not the IMAI case definition, 271 (24%) met IMAI but not SARI, and 327 (28%) did not meet either case definition (Table 3).

**Table 2:** Demographic and clinical characteristics of hospitalized children aged <5 years, (*n* = 4715) and patients aged ≥13 years, (*n* = 1150) with acute respiratory illness at Siaya District Hospital, Kenya, September 2009 to August 2013

Characteristic	Patients <5 years; *N* = 4715 *n* (%)	Patients ≥13 years; *N* = 1258 *n* (%)
Age (years)		
<1	2174 (46·1)	
1–4	2541 (53·9)	
13–17		71 (5·6)
18–49		859 (68·3)
50–64		199 (15·8)
≥65		129 (10·3)
Sex		
Male	2580 (54·7)	445 (35·4)
Female	2135 (45·3)	813 (64·6)
Signs/symptoms		
Measured fever ≥38°C	929 (24·2)	74 (6·4)
Reported fever	4146 (88·0)	873 (69·4)
Cough	4245 (90·1)	995 (79·20)
Difficult breathing	2522 (53·5)	858 (68·3)
Vomiting	2644 (56·1)	706 (56·2)
Convulsions	1095 (23·3)	57 (4·5)
Chest in‐drawing^a^	1120 (23·9)	
Unconscious^a^	633 (15·6)	
Stridor^a^	236 (5·0)	
Nasal flaring^a^	1167 (24·9)	
Unable to drink/breastfeed^a^	527 (11·2)	
Headache^a^		566 (57·1)
Chest pain^a^		548 (43·7)
Lethargy^a^		573 (46·0)
Samples tested	3833 (81·3)	1150 (91·4)
Influenza positive	229 (6·0)	128 (11·1)
Influenza virus types isolated		
Influenza A	157 (68·6)	102 (79·7)
Influenza AH1N1 pandemic	57 (37·8)	33 (40·7)
Influenza AH1N1 seasonal	1 (0·7)	0 (0·0)
Influenza H3N2	30 (19·9)	18 (22·2)
Influenza B	73 (31·9)	27 (21·1)
Influenza A and B	1 (0·4)	1 (0·8)
Influenza unsubtypable	7 (4·6)	6 (7·4)
Not subtyped	56 (37·1)	24 (30·0)

a Variable available for one age category.

**Table 3:** Sensitivity, specificity, predictive value positive (PVP), predictive value negative (PVN), and overlap of different case definitions for the detection of influenza virus infections among hospitalized children aged <5 years (*n* = 3833) and patients aged ≥13 years (*n* = 1150) with acute respiratory illness at Siaya District Hospital, Kenya, 2009–2013

	Case Definition	Influenza positive/total influenza positive	Sensitivity (95% CI)	Specificity (95% CI)	PVP (95% CI)	PVN (95% CI)
Children aged <5 years	SARI (*n* = 2985)	193/229	84·3 (78·9–88·7)	22·5 (21·2–23·9)	6·5 (3·7–10·6)	95·8 (95·0–96·4)
	IMCI Pneumonia (*n* = 2518)	159/229	69·4 (63·0–75·3)	34·5 (33·0–36·1)	6·3 (5·4–7·3)	94·7 (93·3–95·8)
Adolescents and adults aged ≥ 13 years	SARI (*n* = 552)	77/128	60·2 (51·1–68·7)	53·5 (50·4–56·6)	14·0 (8·6–21·3)	91·5 (89·6–93·1)
	IMAI pneumonia (*n* = 627)	73/128	57·0 (48·0–65·7)	45·8 (42·7–48·9)	11·6 (6·7–18·6)	89·5 (87·4–91·3)
Overlap between IMCI versus SARI and IMAI versus SARI						
Children aged <5 years		Number (%) of cases meeting SARI and IMCI Pneumonia Case Definitions	Number (%) of cases meeting just SARI Case Definition	Number (%) of cases meeting just IMCI Pneumonia Case Definition	Number (%) of cases not meeting either of the SARI or IMCI pneumonia case definitions	
		1998 (52·1)	987 (25·8)	520 (13·5)	328 (8·6)	
Adolescents and adults aged ≥13 years		Number (%) of cases meeting SARI and IMAI Pneumonia Case Definitions	Number (%) of cases meeting just SARI Case Definition	Number (%) of RI cases meeting just IMAI Pneumonia Case Definition	Number (%) of RI cases not meeting either of the SARI or IMAI pneumonia case definitions	
		356 (30·9)	196 (17·1)	271 (23·6)	327 (28·4)	

Among the 2985 (78%) patients aged <5 years who met the SARI definition, 193 (7%) were positive for influenza. Of the 2518 (66%) children aged <5 years with IMCI pneumonia, 159 (6%) tested positive for influenza. Of the 552 (48%) patients aged ≥13 years who met the SARI case definition and the 627 (55%) who met the IMAI pneumonia case definition, 77 (14%) and 73 (12%), respectively, tested positive for influenza virus.

In the <5‐year‐old group, SARI had a sensitivity of 84% (95% CI 79‐89) and specificity of 23% (95% CI 21‐24) for laboratory‐confirmed influenza. IMCI pneumonia had lower sensitivity, [69% (95% CI 63‐75)], but higher specificity [35% (95% CI 33‐36)]. The PVP was low and similar for both definitions (Table 3). In adolescents and adults, the sensitivity of SARI of 60% (95% CI 51‐69) was similar to that of IMAI [57% (95% CI 48‐66)]. However, specificity was higher for SARI [54% (95% CI 50‐57)] compared to IMAI [46% (95% CI 43‐49)]. The two case definitions for patients aged ≥13 years had similar PVPs and PVNs (Table 3).

**Figure 1:**
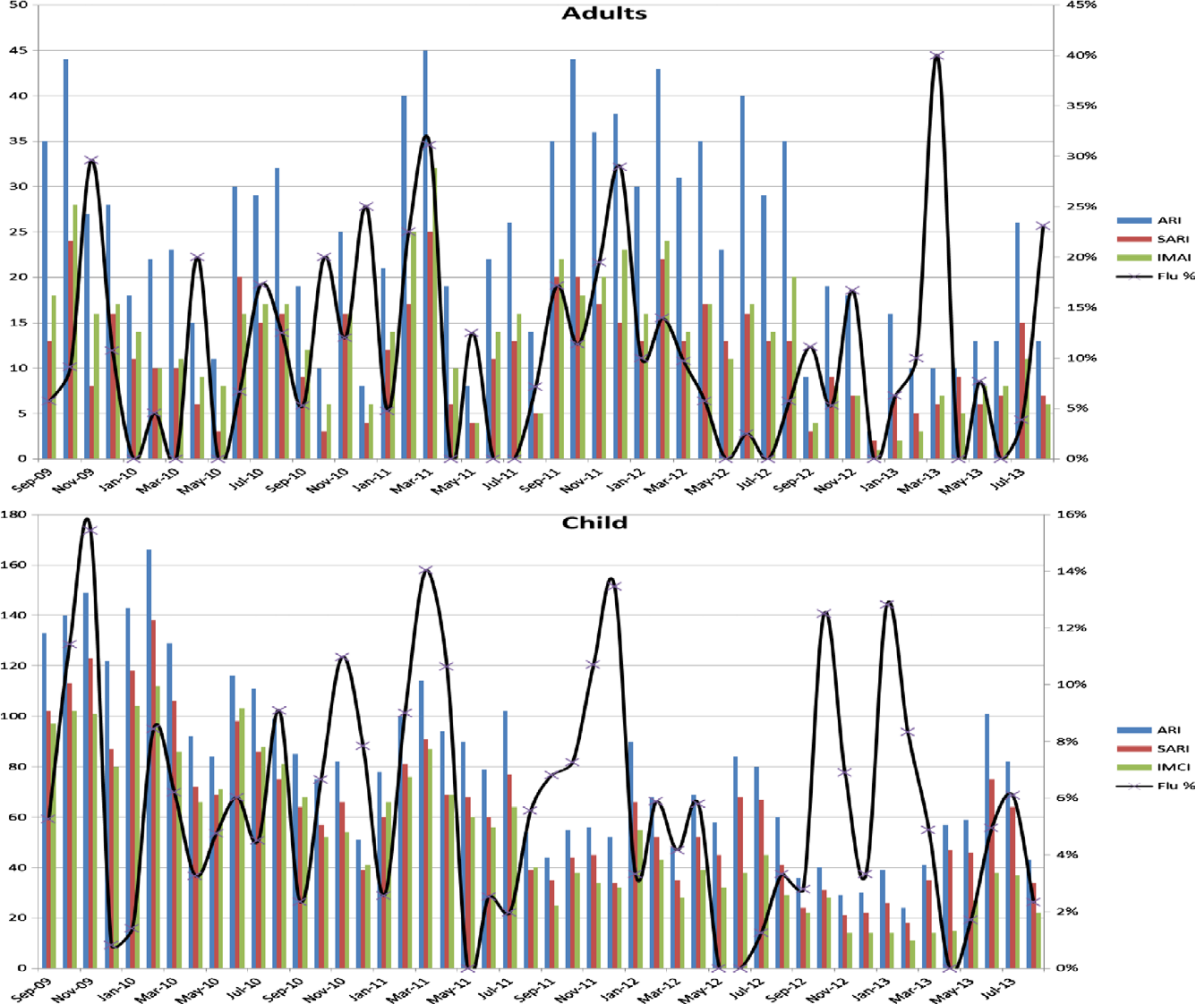
Temporal patterns of Influenza virus activity, ARI and performance of case definitions among ARI hospitalixed patients at SCRH, 2009–2013.

During the study period, influenza circulated year‐round. The highest number of ARI admissions and influenza cases occurred between September and March; the case definitions followed the same disease trends for both age groups (Figure 1).

## Discussion

In our analysis of surveillance data of patients with hospitalized respiratory illness in Kenya, we found that the performance of the WHO case definitions for pneumonia was generally similar to that of the SARI case definition for the detection of laboratory‐confirmed influenza in adolescents and adults. For children aged <5 years, SARI had higher sensitivity, while the IMCI pneumonia case definition had higher specificity. All three case definitions for the two age groups were more sensitive than specific. This balance between sensitivity and specificity would serve the purposes of routine surveillance, case finding for detection of new viral strains and identification of start of an epidemic,^17^ although in surveillance systems with limited resources, more specific case definitions that maximize case detection may be preferred. Temporal analysis of the case definitions (Figure 1) showed that for both age groups, the use of any of the three case definitions would be effective in demonstrating the trends of influenza activity over time—an important objective of most influenza surveillance systems.^3^

A surveillance system that utilizes either the SARI or the pneumonia case definition for inpatient influenza surveillance would likely have the potential to meet most of the objectives of influenza surveillance as outlined in WHO's guidance document on Global Epidemiologic Standards for Influenza (Table 4).^3^ The more widespread use of the familiar IMCI/IMAI pneumonia guidelines for influenza surveillance in resource‐poor countries—where healthcare personnel are limited, healthcare systems are overburdened, 18 and influenza seasonality is less discrete 2—could allow for the integration of hospital‐based influenza surveillance into broader pneumonia surveillance platforms that may be more sustainable. It could also allow for influenza burden and epidemiology to be described in the context of a more familiar clinical and public health syndrome—pneumonia—that resonates widely with global health policymakers like the Gates Foundation and GAVI,^5^, ^6^ dovetails with the UN's Millennium Development Goals 19 and is more easily recognizable to clinicians.^8^

**Table 4:** Objectives of WHO's global epidemiological surveillance standards for influenza

Specific goal: Provide timely and high‐quality epidemiological data and viral isolates to perform the following set of functions:	
1	Describe seasonality of influenza where feasible
2	Signal the start and end of influenza season
3	Provide candidate viruses for vaccine production
4	Describe the antigenic character and genetic make‐up of circulating viruses
5	Identify and monitor groups at high risk of severe disease and mortality
6	Establish baseline levels of activity for influenza and severe influenza‐related disease with which to evaluate the impact and severity of each season and of future pandemic events
7	Generate influenza data that can be used during focused studies to estimate influenza burden and help decision makers prioritize resources and plan public health interventions
8	Identify locally circulating virus types and subtypes and their relationship to global and regional patterns
9	Assist in developing and understanding of relationship of virus strains to disease severity
10	Monitor antiviral activity
11	Detect unusual and unexpected events such as outbreaks of influenza outside the typical season, severe influenza among healthcare workers, or clusters of vaccine failures that may herald novel influenza virus

In addition, by producing baseline data, surveillance systems may also provide a platform for evaluation of vaccine and other intervention effectiveness. Not all of these objectives will be accomplished by every system, particularly when resources are limited (taken from the WHO's Global Epidemiological Surveillance Standards for Influenza, 2013).

Furthermore, pneumonia surveillance for influenza appears to be practical; two recent large studies—one in children and one in adults—demonstrated the ability of inpatient surveillance systems for pneumonia to detect laboratory‐confirmed influenza.^20^, ^21^ While these studies relied on radiographically confirmed pneumonia, they detected influenza in rates similar to what we found in our analysis using IMCI and IMAI case definitions: 7% in children 21 and 6% in adults.^20^ In addition to these studies, global multicenter studies such as the Pneumonia Etiology Research for Child Health Study have used pneumonia case definitions to assess for influenza‐associated pneumonia across multiple sites.^22^

The SARI case definition was more sensitive in capturing cases among children aged <5 years. For this reason, surveillance systems that are interested in estimating influenza‐associated burden of disease in children aged <5 years would arrive at a closer estimate using the SARI case definition. Neither the SARI nor the pneumonia case definition would likely capture the true extent of influenza burden, which can include disease presentations that reflect worsening of preexisting chronic disease rather than simple respiratory illness.^23^

This study had limitations, we tested proportionately fewer ARI cases that died, and therefore, our results may not be representative of all hospitalized ARI. In addition, we did not include infants aged <2 months and children aged 5‐12 years in this analysis because WHO clinical guidelines and case definitions for pneumonia in these age groups are unavailable. This limitation would be a challenge to any surveillance system that chooses to use the WHO case definitions, which would need to be expanded in order to include this age group.

Increasing the use of WHO pneumonia case definitions for influenza surveillance could be an effective and sustainable tool to monitor influenza activity while using a terminology and disease syndrome familiar to both clinicians and policymakers. It could also be a relevant complement to the use of the SARI case definition in children aged <5 years in surveillance systems that wish to more closely approximate burden of disease. Additional analyses from tropical and temperate countries would be helpful in determining whether the performance of SARI, IMCI, and IMAI is similar in other settings.

## Declaration of interest

None.
